# Broccoli Florets Supplementation Improves Insulin Sensitivity and Alters Gut Microbiome Population—A Steatosis Mice Model Induced by High-Fat Diet

**DOI:** 10.3389/fnut.2021.680241

**Published:** 2021-07-28

**Authors:** Gil Zandani, Sarit Anavi-Cohen, Nina Tsybina-Shimshilashvili, Noa Sela, Abraham Nyska, Zecharia Madar

**Affiliations:** ^1^The Faculty of Agriculture, Food and Environment, The Hebrew University of Jerusalem, Rehovot, Israel; ^2^Peres Academic Center, Rehovot, Israel; ^3^Department of Plant Pathology and Weed Research, Volcani Center, Rishon LeZion, Israel; ^4^Sackler School of Medicine, Tel Aviv University, Tel Aviv, Israel

**Keywords:** broccoli, NAFLD, gut microbiome, insulin sensitivity, lipid metabolism, high-fat diet

## Abstract

Nonalcoholic fatty liver disease (NAFLD) is linked to obesity, type 2 diabetes, hyperlipidemia, and gut dysbiosis. Gut microbiota profoundly affects the host energy homeostasis, which, in turn, is affected by a high-fat diet (HFD) through the liver-gut axis, among others. Broccoli contains beneficial bioactive compounds and may protect against several diseases. This study aimed to determine the effects of broccoli supplementation to an HFD on metabolic parameters and gut microbiome in mice. Male (7–8 weeks old) C57BL/J6 mice were divided into four groups: normal diet (ND), high-fat diet (HFD), high-fat diet+10% broccoli florets (HFD + F), and high-fat diet + 10% broccoli stalks (HFD + S). Liver histology and serum biochemical factors were evaluated. Alterations in protein and gene expression of the key players in lipid and carbohydrate metabolism as well as in gut microbiota alterations were also investigated. Broccoli florets addition to the HFD significantly reduced serum insulin levels, HOMA-IR index, and upregulated adiponectin receptor expression. Conversely, no significant difference was found in the group supplemented with broccoli stalks. Both broccoli stalks and florets did not affect fat accumulation, carbohydrate, or lipid metabolism-related parameters. Modifications in diversity and in microbial structure of proteobacteria strains, *Akermansia muciniphila* and *Mucispirillum schaedleri* were observed in the broccoli-supplemented HFD-fed mice. The present study suggests that dietary broccoli alters parameters related to insulin sensitivity and modulates the intestinal environment. More studies are needed to confirm the results of this study and to investigate the mechanisms underlying these beneficial effects.

## Introduction

Nonalcoholic fatty liver disease (NAFLD) is one of the major causes of liver disease worldwide (1). The prevalence of NAFLD in the general population is about 25% and is still increasing (2). NAFLD is a broad term, describing several liver pathologies, ranging from simple hepatic steatosis that is considered benign through steatohepatitis, fibrosis, and, finally, cirrhosis (3). NAFLD is a multisystem disease associated with obesity, insulin resistance, type 2 diabetes mellitus, hyperlipidemia, and metabolic syndrome (2). Microbiota-derived bioactive transition through the gut-liver axis seems to play a pivotal role in the development, progress, and severity of NAFLD (4).

Several factors, inherited as well as acquired, have been implicated in the pathogenesis and pathophysiology of NAFLD, including genetic diversity, epigenomic alterations, sedentary lifestyle, and prolonged positive energy balance (5). However, despite extensive ongoing research, the exact mechanisms are still obscure and most likely involve interactions between multiple organs and tissues (6). Numerous studies highlight the fundamental role of balanced nutrition in NAFLD prevention and treatment (5, 7). Despite the complexity and the high variability that exists between patients with NAFLD (8). healthy diets remain the first line of treatment for NAFLD. Recently, the integration of herbal bioactive molecules and medicinal plants has revealed their beneficial role along with lifestyle interventions in NAFLD treatment (9).

Brassica vegetables belong to the Cruciferous family. These include several popular vegetables like kale, cabbage, cauliflower, and broccoli. They contain trace amounts of fat and are rich in vitamins, minerals, and fibers, as well as many phytochemicals (10). Broccoli is known for its beneficial properties (11), which are usually attributed to its high glucosinolates (especially glucoraphanin) and isothiocyanates (mainly sulforaphane) content (10, 12, 13). Previous data indicated sulforaphane can modulate lipid metabolism and render the antioxidant capacity *in vivo* and *in vitro* (14). The metabolic advantages elicit by glucoraphanin, the precursor of sulforaphane, are also supported. Glucoraphanin has been shown to efficiently affect parameters that are related to obesity, insulin resistance, and NAFLD (15). *In vivo* models, along with human studies, show that glucoraphanin administration improves the lipid profile, fat metabolism, gut microflora composition, and inflammatory factors (16, 17). However, one of the drawbacks of interpreting the results of these studies to an everyday diet is the fact that these studies utilized purified or semipurified compounds instead of whole broccoli (12).

The present study aimed to explore the impact/s of whole (as is) dietary broccoli (florets or stalks) consumption on hepatic glucose and fat metabolism and the consequent changes in the bacterial populations in gastrointestinal microbiota.

## Materials and Methods

### Broccoli Preparation and Analysis

Frozen broccoli florets and stalks were provided by Sunfrost, Israel. Florets and stalks (10% by weight) were grounded into homogenic mash before being added to the experimental diets. Broccoli analysis of macronutrients, fiber, and moisture is presented in Supplementary Table 1.

### Experimental Animals and Diets

Male C57BL/6J mice, 7–8 weeks old, were purchased from Harlan Laboratories (Jerusalem, Israel). All animal care and experimental protocols were performed within the guidelines of the Authority for Biological and Biomedical Models, and were approved by the Institutional Animal Care Ethics Committee, both of the Hebrew University of Jerusalem (AG-19-15838-3). Mice were housed under standardized conditions for animal facilities: controlled environment (12-h light/dark cycle, 18–24°C room temperature). All mice had free access to food and water. After an adaptation period, 32 mice were randomly divided into four groups (*n* = 8): (1) the mice fed a normal diet (ND), (2) the mice fed a high-fat (60%) diet (HFD), (3) the mice fed a high-fat diet + 10% broccoli florets (HFD + F), and (4) the mice fed a high-fat diet + 10% broccoli stalks (HFD + S) for 17 weeks. Pelleted form of each of those diets was prepared by mixing water into the powdered diet. The pellets were air-dried and were stored at −20°C. Fresh diets were provided weekly. Body weight and food intake were monitored one time a week. Diet compositions are presented in Supplementary Table 2.

### Oral Glucose Tolerance Test

Oral glucose-loading tests were performed at week 15 of the experimental period. Prior to the OGTT, the mice fasted for 12 h, and then were weighed and marked. At time 0, an initial baseline glucose measurement was taken. The mice were then given D-glucose (3-g/kg body weight) *via* gavage. Glucose levels in the blood samples were obtained from tail veins and measured at 30, 60, and 120 min after glucose loading with a glucometer (handheld Optimum XceedGlucometer, Abbott Diagnostic Care Ltd.).

### Homa-IR

The insulin resistance index was estimated by the homeostasis model assessment (HOMA) parameter, using the following equation: HOMA = fasting serum insulin (μU/ml) × fasting plasma glucose (mM)/22.5 (18).

### Animal Sacrifice and Organ Collection

At the end of the experiment, the mice fasted overnight, their body weight was recorded, and they were sacrificed in a random order by isoflurane (Minard Inc., USA) anesthesia. Blood was collected from the vena cava, centrifuged at 8,000 rpm at 4°C for 10 min, and stored at −80°C. Adipose tissue was removed, weighed, placed in liquid nitrogen, and stored at −80°C. Liver tissue was collected and weighed. A small sample from the right liver lobe was placed in 4% formaldehyde, and the remaining liver tissue was minced in liquid nitrogen and stored at −80°C. The ceca were separated from the large intestines, and their contents were collected for microbiota analysis.

### Biochemical Analysis of Serum Parameters

Liver enzymes, such as serum alanine aminotransferase (ALT), alkaline phosphatase (ALP), and serum aspartate aminotransferase (AST), were measured by an automated clinical chemistry analyzer along with total cholesterol, high density lipoprotein (HDL), and total triglycerides (American Laboratories Ltd., Herzliya, Israel). Concentrations of plasma insulin were determined by a Rat/Mouse Insulin ELISA Kit (Cat #EZRMI-13K), supplied by Merck.

### Liver Histology Examination

Histological slides were prepared by Patholab (Rehovot, Israel). Livers were macro-dissected, placed in plastic cassettes, and dehydrated. The dehydrated samples were embedded in paraffin blocks by an automatic apparatus. Serial sections 3–5-μm thick were cut from each block, placed on glass slides, stained with hematoxylin and eosin (H and E), and covered by an automatic apparatus. The histopathological examinations were performed by Dr. Abraham Nyska, DVM, Dipl. ECVP, Fellow IATP, board certified in toxicologic pathology—https://ebvs.eu/colleges/ECVP/members/prof-abraham-nyska. Histopathological changes were described and scored by the study pathologist, using semiquantitative grading of five grades (0–4), taking into consideration the severity of the changes. The scoring reflects the predominant degree of the specific lesions seen in the entire field of the histology section. A generic grading criterium was used: (19) zero (0) = no lesion; 1 = minimal change; 2 = mild change; 3 = moderate change; and 4 = marked change.

### Hepatic Lipid Extraction

Quantification of total lipids in the liver was carried out, using the Folch method (20). Frozen liver tissue (100 mg) was homogenized with 700-μL methanol. Then, 1,400 μL of chloroform was added (2:1 v/v) and lightly shaken overnight to separate the two phases. On the next day, the vials were centrifuged at 3,000 rpm for 10 min at room temperature. The upper aqueous phase was removed, and the lower organic phase (containing the lipids) was transferred to a clean, previously weighed tube. The samples were evaporated until complete dryness, measured to determine the lipid fraction weight, and normalized to initial tissue sample weight.

### Western Blot Analysis

Liver tissues were lysed with a lysis buffer, containing 20-mM Tris-HCl (pH 7.4), 145-mM NaCl, 10% glycerol, 5-mM EDTA, 1% Triton X-100,.5% NP-40, 100-mM phenylmethylsulfonyl fluoride (PMSF), 200-mM NaVO4, 5-mM NaF, and 1% protease inhibitor cocktail. Lysates were centrifuged at 14,000 rpm for 15 min, and the protein concentration was determined by the Bradford method, using bovine serum albumin as a standard. The samples were then separated by SDS-PAGE gel (10%) and transferred to nitrocellulose membranes. Blots were respectively incubated with dilutions of primary antibodies: (AMPK 1:5,000, #2532 Cell Signaling Technology; p-AMPK 1:5,000, #2531 Cell Signaling Technology; ACC 1:1,000, #3662 Cell Signaling Technology; p-ACC 1:1,000 #3661 Cell Signaling Technology; AKT 1:5,000 #9272 Cell Signaling Technology; p-AKT 1:1,000, #9271 Cell Signaling Technology; β actin 1:1,000, #3700 Cell Signaling Technology; CD36 1:1,000, ab124515 ABCAM) at 4° overnight. After several washes, the membranes were incubated with a secondary goat antibody (Jackson Immuno-Research Laboratories, West Grove, PA, USA). The immune reaction was detected by enhanced chemiluminescence, with bands being quantified by densitometry and expressed as arbitrary units. β-actin was used as a control protein.

### Quantitative Real-Time PCR

Total RNA was isolated from liver tissues by using Tri-Reagent (Sigma-Aldrich, Rehovot, Israel), according to the protocol of the manufacturer. Complementary DNA was prepared with the High-Capacity cDNA Reverse Transcription Kit (Quanta BioSciences, Gaithersburg, MD, USA). Real-time polymerase chain reaction (PCR) was performed using the 7300 Real-Time PCR System (Applied Biosystems, Foster City, CA, USA), with specific primers as follows: glucose 6-phosphatase (G6pase); phosphoenolpyruvatecarboxykinase (PEPCK); fatty acid synthase (Fasn); peroxisome proliferator-activated receptor alpha (PPARα); sterol regulatory element-binding protein 1c (SREBP-1c); peroxisome proliferator-activated receptor gamma coactivator 1-alpha (PGC-1α); adiponectin receptor 1 (AdipoR1); adiponectin receptor 2 (AdipoR2). Quantitative changes in gene expression were determined by normalizing to 18S. The primer sequences are listed in Supplementary Table 3.

### Gut Microbiota Analysis

The effects of each diet on the bacterial population in the gut microbiome were examined by analyzing the prokaryotic 16S ribosomal RNA gene (16S rRNA), which is approximately 1,500-bp long and contains nine variable regions interspersed among conserved regions. These variable regions were subjected to phylogenetic classification, in diverse microbial populations.

The following protocol describes a two-step PCR-based method for preparing samples for sequencing the variable V3 and V4 regions of the 16S rRNA gene. Bacterial DNA was extracted from fecal samples by using the DNeasyPowersoil kit (Qiagen) according to the instructions of the manufacturer. Each sample was then quantified with a Qubit 2.0 Fluorometer (ThermoFisher Scientific, Waltham, MA, USA) and diluted to a final concentration of 5 ng μL−1 in 10-mM Tris at pH 8.5. The 16S library preparation was carried out as described in Illumina's 16S sample preparation guide with minor modifications. PrimeStar HS DNA Polymerase Premix (Takara-Clontech, Mountain View, CA, USA) was used instead of the PCR enzyme. Sequences with 97% similarity were assigned to the same operational taxonomic units (OTU). OTUs of representative sequences at a similarity of 97% and their relative abundances were used to calculate and analyze rarefaction curves. Bacterial richness and diversity within samples were classified by α diversity (Pielou's index, observed-species indices, and Shannon index).

### Statistical Analysis

Results are presented as mean ± SEM. Data were analyzed by the JMP 14 Pro software suites (SAS Institute, Cary, NC, USA). Comparisons between groups were made by one-way analysis of variance (ANOVA), followed by a Tukey–Kramer test or by unpaired two-tailed Student's T-test. Statistical significance was defined at *p* < 0.05.

## Results

### Effects of Dietary Broccoli on Body and Tissue Weight and Food Intake

Consumption of an HFD led to a significant increase in body weight, epididymal fat, and liver weight (Table 1). These parameters were equally elevated in all HFD-fed groups, regardless of whether the diet was supplemented with broccoli (florets or stalks) or not. Consistent with the alterations in body weight, food intake was similar in the HFD-fed groups (Table 1).

**Table 1:** Effects of dietary broccoli on body weight, food intake, and tissues weight.

	**Group**			
	**ND**	**HFD**	**HFD + F**	**HFD + S**
Initial body weight (g)	19.79 ± 0.60^a^	19.60 ± 0.34^a^	19.72 ± 0.42^a^	20.39 ± 0.29^a^
Final body weight (g)	32.12 ± 1.64^b^	42.19 ± 1.33^a^	43.34 ± 1.56^a^	43.95 ± 1.67^a^
Food intake (g/day)	3.44 ± 0.23^a^	2.71 ± 0.14^b^	2.83 ± 0.16^b^	2.75 ± 0.21^b^
Liver tissue weight (g)	1.09 ± 0.08^b^	1.76 ± 0.03^a^	1.77 ± 0.19^a^	1.78 ± 0.19^a^
Adipose tissue weight (g)	1.16 ± 0.15^b^	1.66 ± 0.07^a^	1.82 ± 0.13^a^	1.85 ± 0.06^a^

*Male C57BL/6J mice were fed with normal diet (ND), high-fat diet (HFD), HFD + 10% broccoli florets (HFD + F), HFD + 10% broccoli stalks (HFD + S) for 17 weeks. Values are expressed as mean ± SEM (n = 8). Means without a common letter are statistically different (p < 0.05)*.

### Broccoli Enrichment Affects Parameters Related to Glucose Homeostasis

**Figure 1:**
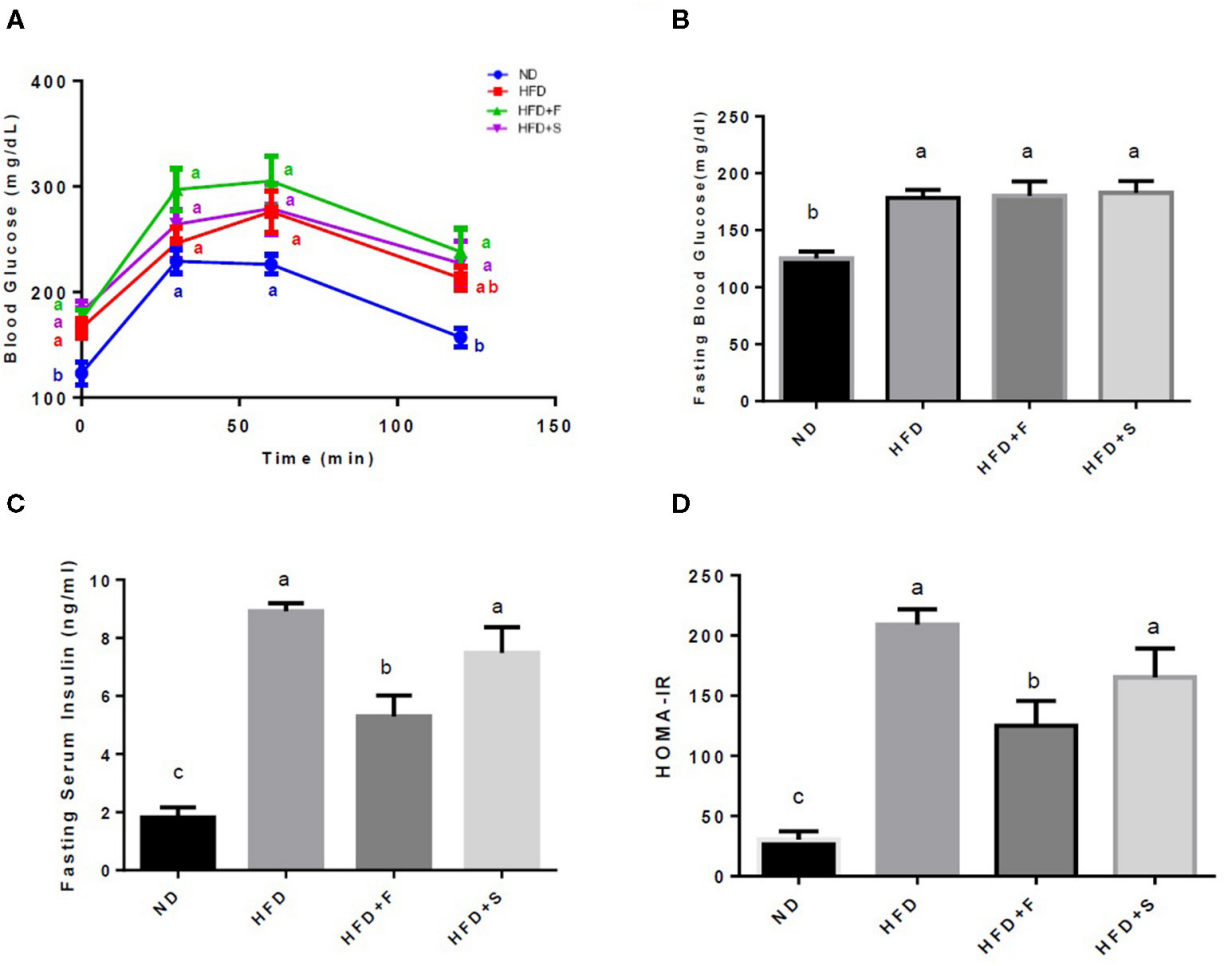
Effects of broccoli enrichment on glucose homeostasis. Male C57Bl/6J mice fed a normal diet (NO), high fat diet (HFD), HFD + l0% broccoli florets (HFO + F), HFD + 10% broccoli stalks (HFD + S) for 17 weeks. Oral glucose tolerance test (120 min duration) was performed at week 15 **(A)** Mean fasting blood glucose concentration at sacrifice **(B)** insulin serum levels at sacrifice **(C)** and homeostatic model assessment of insulin resistance index **(D)**. Values are expressed as mean ± SEM (*n* =7–8). Means without a common letter are statistically different.

A glucose tolerance test was performed on the 15th week of the feeding period. Along the entire OGTT, basal blood glucose was markedly elevated in the HFD-fed groups. The ND group exhibited significantly lower glucose levels compared with the other groups, while all HFD-fed mice showed no significant differences at any of the time points (Figure 1A). At the end of the diet regime, fasting blood glucose and serum insulin levels were determined, and the HOMA-IR index was calculated thereafter.

All the HFD-fed groups exhibited increased glucose blood levels in comparison with the ND group. However, blood insulin levels were significantly lower in the group that was supplemented with broccoli florets (Figures 1B,C). This resulted in lower HOMA-IR values in the broccoli florets supplemented group (Figure 1D).

### Effects of Broccoli Supplementation on the Serum Lipid Profile, Liver Enzymes, Lipid Content, and Liver Histology

Table 2 demonstrates the changes in serum lipids levels. Total triglyceride levels of all HFD-fed groups were profoundly lower than in the ND group, whereas a reversed pattern was observed for total blood cholesterol and HDL levels.

**Table 2:** Effects of broccoli supplementation on the serum lipid profile and liver enzymes.

	**Group**			
	**ND**	**HFD**	**HFD+F**	**HFD+S**
**Lipid profile**				
Total triglycerides (mg/dL)	136.00 ± 17.48^a^	81.87 ± 5.54^b^	80.50 ± 4.15^b^	81.00 ± 3.25^b^
Total cholesterol (mg/dL)	152.60 ± 15.64^b^	234 ± 33.84^a^	214.25 ± 13.14^a^	220.00 ± 12.58^a^
HDL (mg/dL)	122.10 ± 10.35^b^	170.93 ± 12.83^a^	165.68 ± 14.15^a^	163.00 ± 9.57^a^
**Liver enzymes**				
ALP (IU/L)	76.60 ± 2.18	68.87 ± 4.82	69.25 ± 5.07	68.00 ± 3.95
AST/SGOT (IU/L)	69.25 ± 7.69^b^	110.63 ± 13.86^ab^	146.25 ± 17.04^a^	88.00 ± 11.39^b^
ALT/SGPT (IU/L)	40.00 ± 8.50^b^	73.42 ± 9.95^b^	131.00 ± 15.83^a^	82.00 ± 13.08^b^

*Male C57BL/6J mice were fed with normal diet (ND), high-fat diet (HFD), HFD + 10% broccoli florets (HFD + F), HFD + 10% broccoli stalks (HFD + S) for 17 weeks. Values are expressed as mean ± SEM (n = 8). Means without a common letter are statistically different (p < 0.05)*.

Serum ALP levels did not show intergroup differences. Compared with the control, AST and ALT levels were statistically higher only in the broccoli-florets-added group, while their levels did not differ in the other HFD-fed groups.

**Figure 2:**
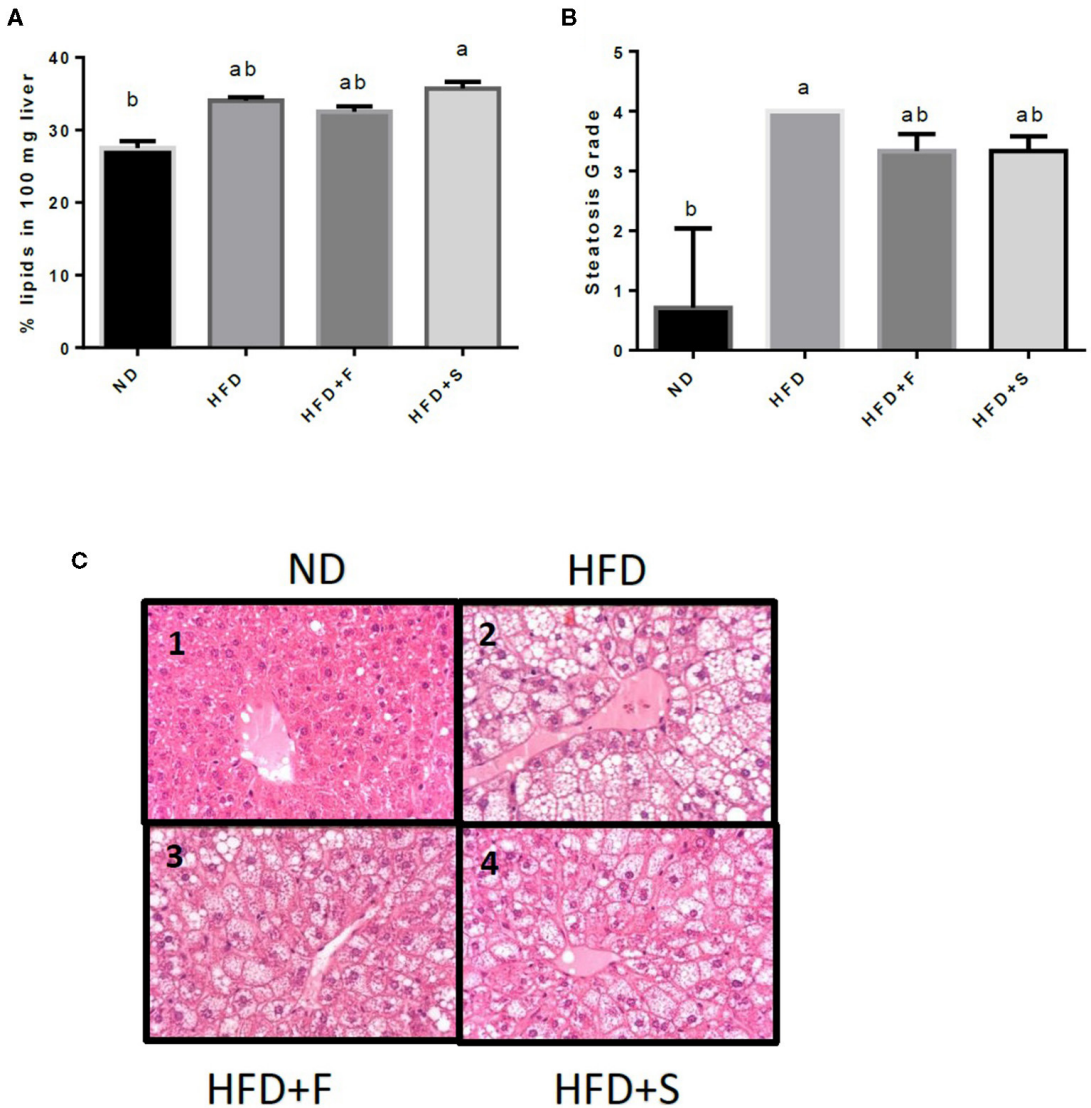
Effects of broccoli consumption on lipid content and liver histology Male CS7BL/6J mice fed a normal diet (ND), high fat diet (HFD), HFD + 10% broccoli florets (HFD + F), HFD + l0% broccoli stalks (HFD + S) for 17 weeks. Evaluation of lipid content was performed by using the Folch method **(A)** histologocal evaluation of steatosis grade **(B)** Histology section of the penlobular region of the lover by H&E stain (**C**, l−4). Values are expressed as mean ± SEM (*n* = 4–8). Means without a common letter are statistocally different (*p* < 0.05).

Liver fat accumulation appeared to increase in all the HFD-fed groups, but only the HFD+S reached significance (Figure 2A). The results were further substantiated by liver histology H and E staining results that were approximately similar to those obtained by the Folch method, although, in this analysis, only the HFD group significantly differed from the control (Figures 2B,C).

### Effect of Diets on Markers Involved in Carbohydrate and Fat Metabolism in the Liver

**Figure 3:**
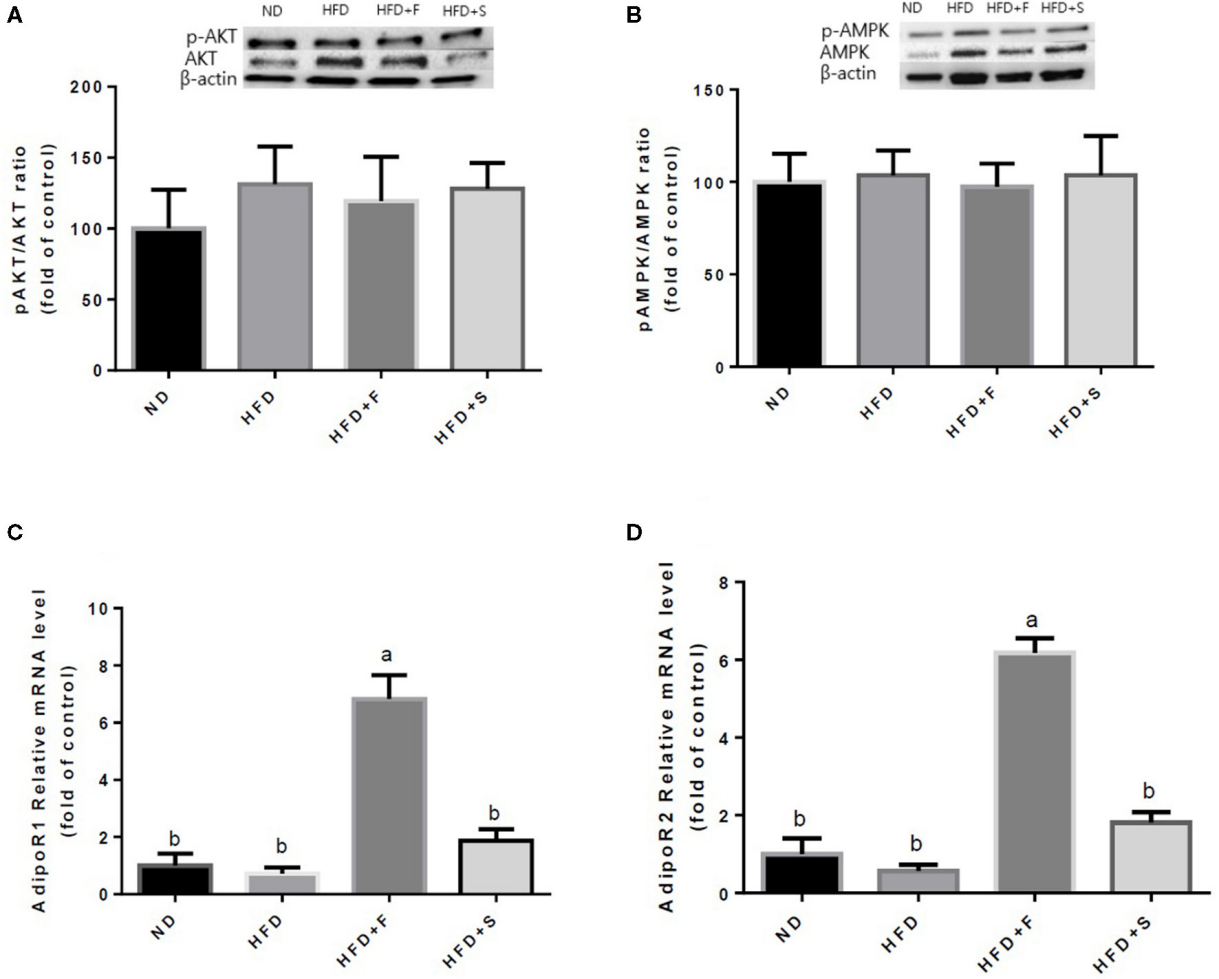
Effect of broccoli supplementation on markers involved in carbohydrates and fat metabolism in the liver. Male C57Bl/6J mice fed a normal diet (ND), high fat diet (HFD), HFD + 10% broccoli florets (HFD + F), HFD + 10% broccoli stalks (HFD + S) for 17 weeks. Protein levels and gene expression of p-AKT/AKT **(A)** p-AMPK/AMPK **(B)** AdipoRl **(C)** and AdipoR2 **(D)** were measured. Values are expressed as mean ± SEM. Means without a common letter are statistically different (*p* < 0.05).

**Figure 4:**
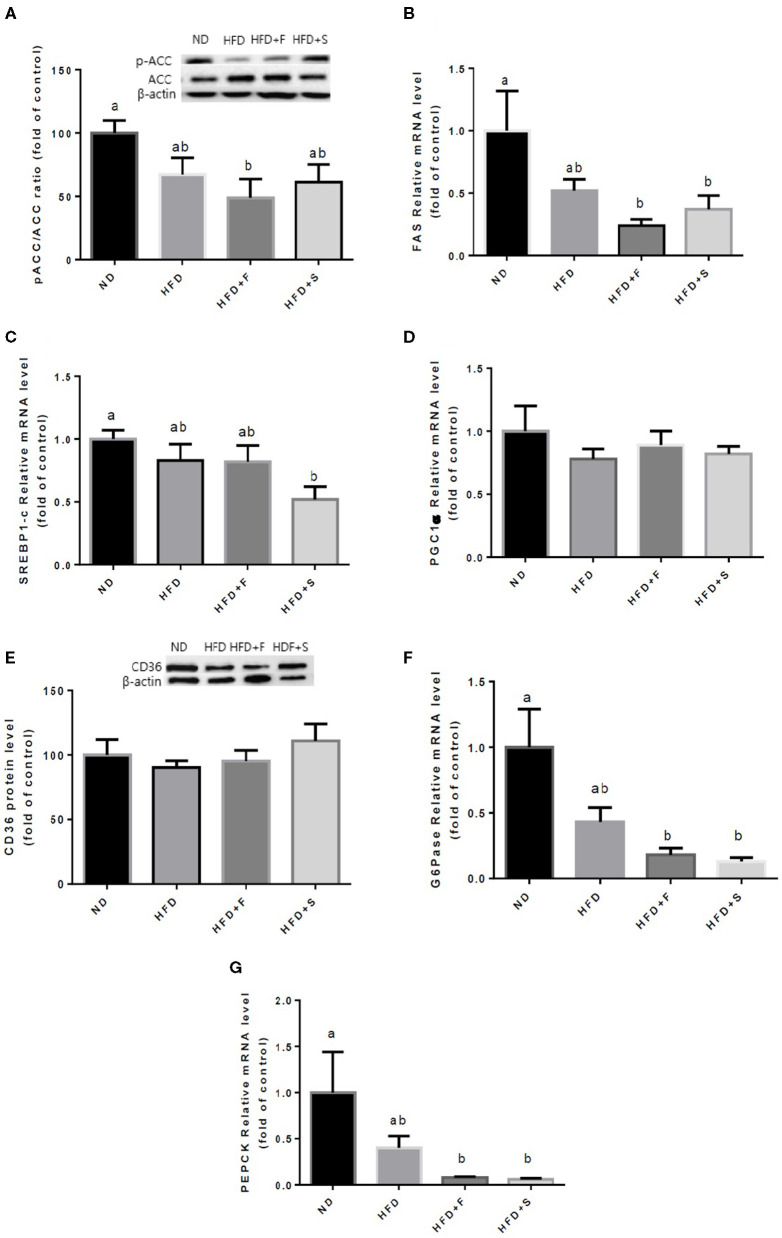
Effect of broccoli supplementation on markers involved in liver fat metabolism. Male C57BL/6J mice fed a normal diet (ND), high fat diet (HFD), HFD + 10% broccoli florets (HFD + F), HFD + 10% broccoli stalks (HFD + S) for 17 weeks. Protein levels and genes expression of p-ACC/ACC **(A)** FAS **(B)** SREBPl-c **(C)** PGC-lα **(D)** CD36 **(E)** G6pase **(F)** and PEPCK **(G)** were measured. Values are expressed as mean ± SEM. Means without a common letter are statistically different (*p* < 0.05).

Further analyses were conducted to evaluate the effect of broccoli addition to HFD on key metabolic regulators. No changes were found in AKT and AMPK activation, assessed by the ratio between phosphorylated to total protein (Figures 3A,B). Conversely, the expression of both adiponectin receptors, AdipoR1, and AdipoR2 was upregulated in the HFD+F group (Figures 3C,D). To further elucidate the metabolic impact of broccoli addition to an HFD, the expression and protein levels of enzymes and factors that participate in hepatic glucose and fat metabolism were examined. p-ACC (ser 79) to the ACC ratio is a surrogate measure for ACC inhibition. Although a tendency toward diminished ACC inhibition was noticed in all HFD-fed groups, this reduction was significantly only in the HFD+F group compared with ND (Figure 4A). Broccoli supplementation (florets or stalks) to HFD negatively affected the expression of FAS (Figure 4B). Consistent with the latter result, lower *de novo* lipogenesis capacity in the HFD+S group was also supported by a decrease in SREBP1-c expression (Figure 4C). PGC-1α expression and CD36 protein levels were unaffected by diet composition (Figures 4D,E). Expression of the key gluconeogenicenzymes, G6pase, and PEPCK was substantially reduced in the two groups that were supplemented with broccoli, compared with the ND group (Figures 4F,G).

### Effect of Dietary Broccoli on Microbiota Richness and Diversity, and Mice Gut Bacterial Community

The interconnection between diet and microbiota composition was assessed at different levels. To evaluate the differences within the samples, the alpha diversity was calculated. The Shannon index, which represents the community diversity and richness, was inferior in the HFD and HFD+S groups compared with that of the control (Table 3). The observed OTU's, which represent the rare species in each group, were found to differ between the HFD + F and the HFD group, with higher levels registered in the former (Table 3). Pielou's index, which indicates species evenness, decreased in the HFD+S group compared with the ND group (Table 3).

**Table 3:** Dietary broccoli impact on microbiota richness and diversity.

	**ND**	**HFD**	**HFD + F**	**HFD + S**
Shannon index	5.98 ± 0.03^a^	5.61 ± 0.05^b^	5.82 ± 0.06^ab^	5.62 ± 0.06^b^
Observed OUTs	124.60 ± 2.29^ab^	112.00 ± 2.65^b^	128.00 ± 3.67^a^	123.00 ± 3.35^ab^
Pielou's index	0.86 ± 0.01^a^	0.82 ± 0.01^ab^	0.83 ± 0.01^ab^	0.81 ± 0.01^b^

*Male C57BL/6J fed with normal diet (ND), high-fat diet (HFD), HFD + 10% broccoli florets (HFD + F), HFD + 10% broccoli stalks (HFD + S) for 17 weeks. Values are expressed as mean ± SEM (n = 5). Means without a common letter are statistically different (p < 0.05)*.

**Figure 5:**
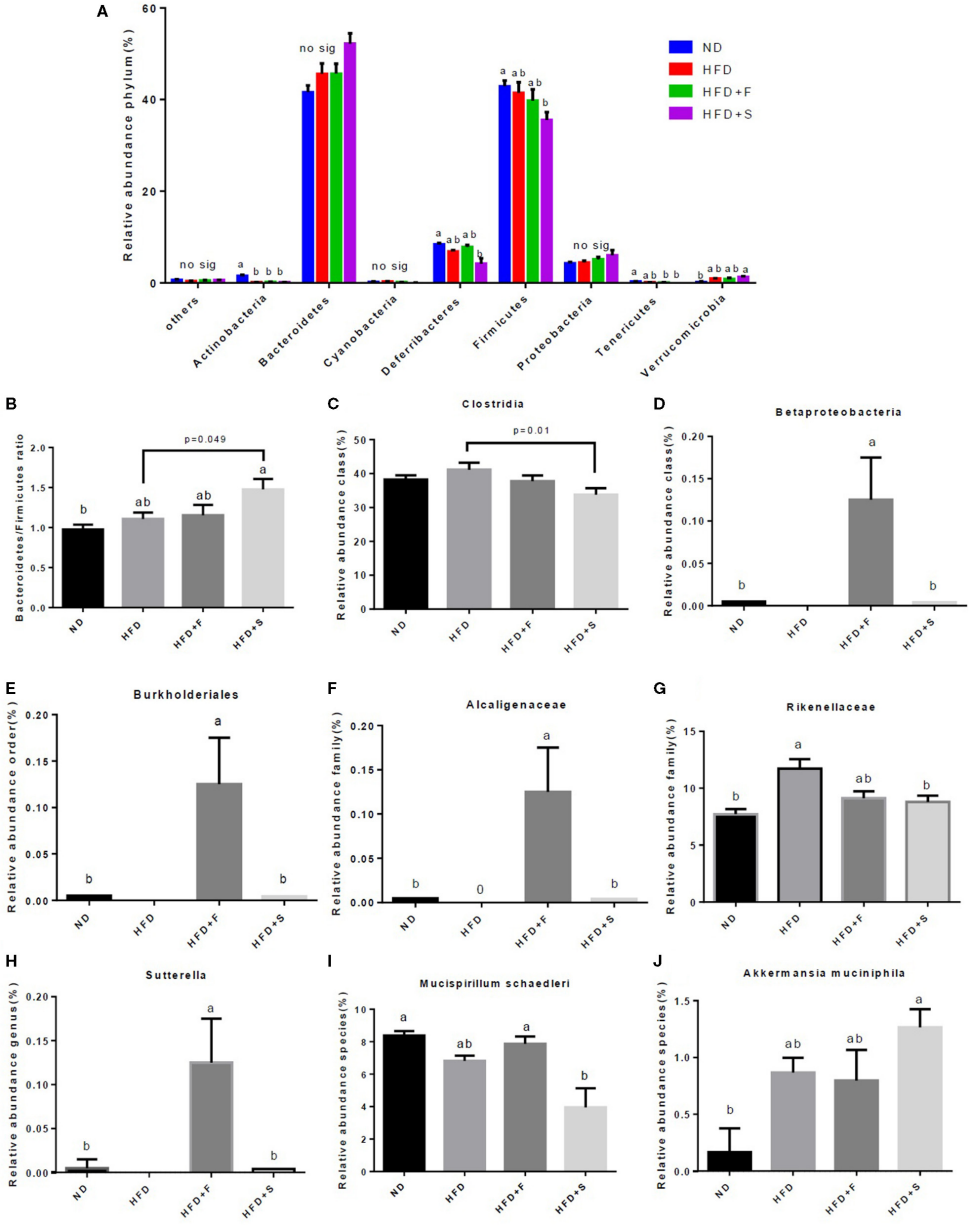
Effect of broccoli supplementation on gut microbiota composition evaluated at all taxonomic levels. Male C57Bl/6J mice fed a nonmal diet (ND), high fat diet (HFD), HFD + 10% broccoli florets (HFD + F), HFD + 10% broccoli stalks (HFD + S) for 17 weeks. Phylum level abundance **(A)** Bacteroidetes/Firmicutes ratio **(B)** Class level abundance **(C,D)** order level abundance **(E)** Family level abundance **(F,G)** Genus level abundance **(H)** and species level abundance **(I,J)**. Values are expressed as mean ± SEM (*n* = 5). Means without a common letter are statistically different (*p* < 0.05).

Microbiota composition, following the treatments, was evaluated at all taxonomic levels. At the phylum level, Actinobacteria abundance was lower in all HFD groups, while Deferribacteres and Firmicutes abundance decreased only in the HFD + S group and that of the Tenericutes in both broccoli-added groups, compared with the ND group (Figure 5A). Conversely, Verrucomicrobia abundance was markedly increased in the HFD + S group compared with the ND group (Figure 5A). The Bacteroidetes/Firmicutes ratio was elevated in the HFD + S group compared with the other groups (Figure 5B). At the class level, Clostridia abundance was significantly lower in the stalks group (Figure 5C), whereas Betaproteobacteria abundance was significantly elevated in mice fed with broccoli florets compared with the ND group (Figure 5D). Bburkholederiales and Sutterella abundances were increased at the order and genus levels, respectively, in the HFD + F group (Figures 5E,H). At the family level, Alcaligenaceae abundance was statistically higher in the HFD + F group compared with the other groups (Figure 5F), while mice that were fed with HFD + broccoli stalks showed a reduction in Rikenellaceae abundance compared with the HFD group (Figure 5G). At the species level, a decrease in the abundance of *Mucispirillum schaedleri* was observed in the HFD + F group compared with the HFD + S group (Figure 5I), while the *Akkermansia muciniphila* community was elevated in the HFD+S group compared with the ND group (Figure 5J).

## Discussion

The present study examined the effects of broccoli supplementation under the conditions of diet-induced liver steatosis. The obtained results demonstrated that, although the addition of broccoli to an HFD did not ameliorate body and tissues weight gain or food intake, it positively improved insulin sensitivity and promoted modifications in gut microbiota composition.

NAFLD is characterized by elevated ALT and AST levels, which reflects nonspecific hepatocellular damage (21). In the present study, serum AST and ALT levels statistically decreased in the stalks-supplemented group compared with the florets group, which exhibited the greatest increase of all the groups. Previous studies showed that broccoli consumption does not affect ALT and AST levels (22, 23). Contreras-Zentella et al. reported that a drastic increase of liver transaminase activities in the serum, which was suggested to be considered as an index marker of hepatotoxicity, does not necessarily reflect liver cell death (24). Vegetables with high concentrations of glucosinolates, such as broccoli, naturally contain goiterogens, which were found to lower thyroid hormones levels (25). A recent study has found a relationship between lower thyroid gland functionality and the severity of liver damage (26). This, possibly, can explain why mice supplemented with broccoli demonstrated higher exacerbation in liver tissue. This might also explain the enigmatic increase in liver enzymes in mice supplemented with broccoli florets.

Elevated liver enzymes in the HFD+F group appear to be independent of liver fat accumulation or the degree of steatosis. Indeed, all the HFD-fed groups demonstrated comparable fat accumulation with no marked effect for broccoli supplementation. Moreover, in the current study, blood TG levels were significantly lower in all the HFD-fed mice, compared with the control. Previous studies conducted in mice with the same or closely related genetic background as this study (B6J and B6N) also reported decreased TG levels, following the consumption of an HFD (27, 28). Although not tested, several explanations may account for this phenomenon, including that HFD consumption induced increased hepatic TG clearance and/or decreased hepatic TG export. Similarly, increased HDL blood levels, following the consumption of an HFD, was also previously observed (29, 30). Nevertheless, HDL levels are not indicators reliable for functionality of this lipoprotein nor the individual atherogenic risk (31).

Liver fat metabolism was not significantly affected by the broccoli addition to the HFD as all the HFD-fed groups did not differ from one another. However, FAS expression tended to decrease, to a greater extent, in both broccoli-supplemented groups and, in contrast to the HFD group, reached significance compared with the control, ND group. A similar pattern was observed in ACC activation and SREBP-1c expression in the HFD+F and HFD+S groups, respectively. It can be speculated that a longer time period would have intensified the outcome of the observed differences in lipogenesis between the groups. Although additional extended work is required, this inference is substantiated by the results of Chen et al., in which broccoli intervention through diet did lead to a reduction in liver weight but, similar to our study, did not reduce final body weight, food intake, or fat mass (23).

Insulin sensitivity is metabolically altered by HFD. Here, HFD consumption triggered an increase in serum insulin concentration and HOMA-IR. However, the addition of broccoli florets profoundly ameliorated HFD-induced insulin resistance (32). The positive effects observed due to broccoli florets addition to the HFD might be related to glucosinolates found in broccoli. Previous studies, which administered isolated bioactive compounds from broccoli, sulphoraphane, or glucoraphanin, demonstrated improvements in insulin resistance and glucose tolerance (16, 33). It is known that glucosinolate content is lower in stems and roots (34). Therefore, reduced concentrations of these secondary metabolites may account for the lack of effect of the stalks supplementation on insulin and glucose metabolism.

Disruptions in insulin signaling are tightly associated with NAFLD (35). Akt is an obligate intermediate for proper insulin signaling and plays a central role in the metabolic axis activated by insulin (36). Basal AKT activation, as determined by its phosphorylation at Ser473, remained unchanged in all groups. Yet, as mentioned above, differences in serum insulin concentration and HOMA-IR were observed with HDF + F. Therefore, it can be argued that higher insulin concentrations were needed in the HFD and HFD + S groups to lead to the same AKT basal phosphorylation as the group that was supplemented with broccoli florets. However, it must be emphasized that, although AKT is a central player in the insulin pathway, insulin signaling does not solely rely on AKT activation, and several other PI3K-dependent and independent nodes participate to create full effect of insulin (37). Thus, other insulin-dependent pathways could have been differently affected by the broccoli addition.

Indeed, inconsistency exists regarding the influence of HFD on gluconeogenic enzymes expression/activity. Whereas short-term chronic exposure to an HFD leads to an increase in gluconeogenesis; (38, 39) others found no such effect (40). In the current work, G6pase and PEPCK, key enzymes involved in gluconeogenesis, exhibited nonsignificant differences between all HFD groups, although a tendency toward lower expression levels in the broccoli supplemented groups was observed. Thus, longer feeding duration and/or higher broccoli content are needed to reach significance. Given that insulin sensitivity was reduced by HFD, decreased PEPCK and G6Pase expression appears to occur independently of insulin.

Another dominant player that can profoundly affect lipid and carbohydrate metabolism, including gluconeogenesis inhibition, and is known to augment insulin sensitivity is AMPK. Following its activation, AMPK promotes energy-producing pathways while it “shuts down” energy-consuming pathways (41). This kinase activation is mediated through several factors, including adiponectin. Although not tested directly in the present study, the ability of broccoli-contained component/s to increase adiponectin levels is in agreement with a previous study conducted in HFD-fed rats, which were supplemented with two different doses of broccoli extract (200 or 400 mg/kg). There, broccoli extract-treated rats presented higher concentrations of serum adiponectin (32).

Adiponectin exerts its effects through binding and activation of its receptors, adipoR1 and adipoR2 (42). Adiponectin downstream effectors in the liver appear to have been induced in the HFD + F group, at least to some extent, as these hormone receptors were upregulated in this group. However, in contrast to the induction of adiponectin receptors by broccoli florets, AMPK activation remained unaffected by the diet consumed, suggesting this kinase is not the link to the metabolic changes observed. Nevertheless, the expression pattern of these receptors was somewhat correlated with insulin sensitivity in the HFD-fed groups. Indeed, AdipoR1 and AdipoR2 were found to regulate insulin sensitivity in insulin target tissues, and are important in the pathophysiology of insulin resistance (43). The role of adiponectin in the observed increase in insulin sensitivity in the HFD + F group remained to be defined.

Emerging evidence provides an argument about the importance of the gut microbiome in influencing host homeostasis. There is a close association between gut microbiota dysbiosis and intestinal disorders. In fact, dysbiosis can be considered as a biomarker of such disorders (44). Recently, dysbiosis has been suggested to play a critical role in promoting metabolic diseases, (45) such as NAFLD and NASH, among others (46).

Consumption of brassica vegetables was linked to alterations in the gut bacterial community (47, 48). This is in accord with the role that is ascribed to diet and different environmental factors in maintaining gastrointestinal health (46) and further emphasizes the specific influence of broccoli.

In the present work, several alpha diversity indexes were evaluated. The Shannon index represents the community diversity and richness, which are related to the ability to withstand external threats (44). Although this index was not affected by broccoli consumption, it was significantly lower in all the HFD-fed groups, suggesting reduced diversity compared with the ND group. HFD feeding has been associated with modifications in the gut microbial profile as well as decreased diversity (49), with most studies demonstrating diminished microbiota diversity, independent of the fat percentage in the diet (% calories) (46). Conversely, in the broccoli floret-supplemented group, we detected an increase in observed OTUs, which represent the rare species in each group. This finding is consistent with previous animal studies, showing that 4 days of broccoli consumption increased the number of OTUs in rats (50, 51), although broccoli supplementation in humans did not affect the alpha diversity indexes (52).

Dietary broccoli induced further alterations in the gut bacterial composition. The diversity of dominant gut bacteria, Firmicutes, and Bacteroidetes was shifted toward a higher Bacteroidetes/Firmicutes ratio in the broccoli stalks group. Previous studies performed on the HFD models reported reduced Bacteroidetes/Firmicutes ratios with an increase in this ratio, indicating anti-obesity effects (53, 54). However, not all works appear to support this trend (55), and, thus, consensus has not been reached.

Other taxonomical levels have also been modified by broccoli consumption. A dramatic increase in Proteobacteria strains (Betaproteobacteria, Burkholederiales, Alcaligenaceae, and Sutterella) was noted, although no significant difference was observed in Proteobacteria phylum abundance. Significant abundance of Betaproteobacteria, Burkholederiales, Alcaligenaceae, and Sutterella was found in a mouse-induced obesity model in comparison with mice supplemented with prebiotics (56). Hiippala et al. (57) showed that the *Sutterella* genus, which has been suggested to have an immunomodulatory role due to the ability to adhere to intestinal epithelial cells, do not contribute significantly to microbiota dysbiosis.

HFD consumption augmented Clostridia and Rikenellaceae abundance, and a reduction in the broccoli stalks group was observed. As previously described, an elevated Rikenellaceae abundance was found in HFD-fed mice (58–60), along with higher levels of the Clostridia class (61, 62). Several works have suggested that elevated Clostridia species may represent a protective factor against allergens, and this elevation also increases IL-22, which improves intestinal permeability (63, 64).

Dietary broccoli also affected bacteria that are related to the intestinal mucus layer. A significant reduction was noticed in *Mucispirillum schaedleri* (Deferribacteres phylum) abundance in the stalks group compared with the florets group. *Mucispirillum schaedleri* is found in low levels in mammalian intestinal microbiota (65). Microbial dominance of *Mucispirillum* was associated with proinflammatory responses in the gut microbiota mucosa (66). However, the genetic and physiological features of this species during inflammation and diseases are still not fully understood (67). *Akkermansia muciniphila* (Verrucomicrobia phylum), which is a mucin-degrading community, was elevated in the group that was supplemented with the broccoli stalks. This bacterium resides in the mucus layer and was reported to ameliorate the host metabolic functions and intestinal immunity (68, 69). An increase in the *Akkermansia* species population was found to reduce low-grade inflammation by suppressing the expression of pro-inflammatory cytokins (70) and by preserving gut integrity (71). Furthermore, higher abundance of *Akkermansia muciniphila* was previously found to be associated with improved glucose metabolism, leading to reduced fasting blood glucose levels (72, 73). However, in the present study, insulin and glucose levels remained unchanged, following the consumption of broccoli stalks.

It is important to emphasize that broccoli is a single vegetable and, generally, not eaten alone. Thus the combination with other ingredients in the diet might substantially affect the outcome (11).

## Conclusions

In summary, addition of broccoli florets to an HFD ameliorated insulin sensitivity manifested by reduced serum insulin and HOMA-IR index. Broccoli florets further promoted gut microbiota diversity and low-grade inflammatory-associated strains. Stalk supplementation also altered gut microbiota, leading to the increased Bacteroidetes/Firmicutes ratio and levels of communities that preserve the mucus layer and gut integrity while simultaneously decreasing the levels of potentially harmful species. Future research is needed to elucidate the roles of dietary broccoli addition in metabolic parameters and to comprehensively define the changes in the gut microbiota and their subsequent outcomes. Also, the bioactive compounds, which may contribute to the described results, required further elucidation.

## Data Availability Statement

Datasets presented in this study can be found in NCBI short reads archive under BioProject accession PRJNA728821.

## Ethics Statement

The animal study was reviewed and approved by the Hebrew University of Jerusalem's guidelines of the Authority for Biological and Biomedical Models, and was approved by its Institutional Animal Care Ethics Committee (AG-19-15838-3).

## Author Contributions

ZM and GZ conceived the study and drafted the manuscript. SA-C, NT-S, NS, and AN contributed to the methodology and data processing. All the authors critically revised the manuscript for intellectual content, approved the final version, and agreed to be accountable for all the aspects of the work.

## Conflict of Interest

The authors declare that the research was conducted in the absence of any commercial or financial relationships that could be construed as a potential conflict of interest.

## Publisher's Note

All claims expressed in this article are solely those of the authors and do not necessarily represent those of their affiliated organizations, or those of the publisher, the editors and the reviewers. Any product that may be evaluated in this article, or claim that may be made by its manufacturer, is not guaranteed or endorsed by the publisher.
