# Supplementary material for: Broccoli Florets Supplementation Improves Insulin Sensitivity and Alters Gut Microbiome Population—A Steatosis Mice Model Induced by High-Fat Diet
Source: Front Nutr. 2021 Jul 28;8:680241. doi: 10.3389/fnut.2021.680241 (PMC8355420; doi:10.3389/fnut.2021.680241)
Supplement: Supplementary file 1 [file Data_Sheet_1.docx]

Supplementary Material

**Supplementary Table 1: Macronutrients, fiber and moisture provided by broccoli:**

| 100 gr broccoli stalks | 100 gr broccoli florets |  |
| --- | --- | --- |
| 3.4 | 4.9 | Carbohydrate (g) |
| 2.87 | 3.36 | Fiber (g) |
| 0.86 | 2.73 | Protein (g) |
| 0.33 | 0.36 | Fat (g) |
| 94.99 | 91.34 | Moisture (g) |
| 5.01 | 8.66 | Dry weight (gr) |
| 20 | 33 | Total energy (Kacl) |

**Supplementary Table 2: Compositions of animal diets**

| High Fat Diet  (HFD) | | Normal Diet  (ND) | |  |
| --- | --- | --- | --- | --- |
| Kcal | gr | Kcal | gr | **Ingredients** |
| 1060 | 265 | 840 | 210 | Casein |
| 16 | 4 | 12 | 3 | L- methionine |
| 0 | 0 | 2000 | 500 | Cornstarch |
| 640 | 160 | 400 | 100 | Dextrose |
| 360 | 90 | 156.6 | 39.15 | Sucrose |
| 2790 | 310 | 0 | 20 | Lard |
| 270 | 30 | 180 | 20 | Soybean oil |
| 0 | 0 | 180 | 20 | Anhydrous milkfat |
| 0 | 65.5 | 0 | 35 | Cellulose |
| 0 | 0 | 0 | 0 | Cholesterol |
| 0 | 0 | 0 | 0 | Cholic acid |
| 0 | 51.4 | 0 | 35 | Mineral mix |
| 0 | 21 | 0 | 15 | Vitamin mix |
| 0 | 3 | 0 | 2.75 | Choline chloride |
| 0 | 0.014 | 0 | 0.014 | BHT |
| 5136 | 1000 | 3769 | 1000 | Total energy (Kacl) |

BHT, butylated hydroxytoluene

**Supplementary Table 3: primers sequences**

| **Name** | **Reverse** | **Forward** |
| --- | --- | --- |
| 18s | 5’-CCTCAGTTCCGAAAACCAAC-3’ | 5'-ACCGCAGCTAGGAATAATGG-3' |
| G6pase | 5’-AAGAGATGCAGGAGGACCAA-3’ | 5’-ACTCCAGCATGTACCGGAAG-3’ |
| PEPCK | 5’-TGCAGGCACTTGATGAACTC-3’ | 5’-CAAACCCTGCCATTGTTAAG-3’ |
| Fasn | 5’-GGTCGTTTCTCCATTAAATTCTCAT-3’ | 5’-CTAGAAACTTTCCCAGAAATCTTCC-3’ |
| PPARα | 5’-CTGCGCATGCTCCGTG-3’ | 5’-CTTCCCAAAGCTCCTTCAAAAA- 3’ |
| Srebp-1c | 5’-TAGATGGTGGCTGCTGAGTG-3’ | 5’-GATCAAAGAGGAGCCAGTGC-3’ |
| PGC-1α | 5’-AGAGCAAGAAGGCGACACAT-3’ | 5’-AACAAGCACTTCGGTCATCC-3’ |
| AdipoR1 | 5’-CACATCTACGGGATGACTCTCCA-3’ | 5’-AGTTCATGTATAAGGTCTGGGAGG-3’ |
| AdipoR2 | 5’-CATGATGGGAATGTAGGAGC-3’ | 5’-TTCCTATTATGAAAATAGCCCGGA-3’ |

18S – 18S ribosomal RNA; G6pase- Glucose 6-phosphatase; PEPCK- Phosphoenolpyruvate; carboxykinase; Fasn- fatty acid synthase gene; PPARα- peroxisome proliferator-activated receptor alpha; SREBP-1c- sterol regulatory element-binding protein 1c; PGC-1α- peroxisome proliferator-activated receptor gamma coactivator 1-alpha; AdipoR1- Adiponectin receptor 1; AdipoR2- Adiponectin receptor 2
